# Gallbladder Carcinoma in a Eurasian Otter (*Lutra lutra*)

**DOI:** 10.3390/ani15172484

**Published:** 2025-08-24

**Authors:** Lorenzo Domenis, Marzia Pezzolato, Elena Biasibetti, Raffaella Spedicato, Serena Robetto

**Affiliations:** Istituto Zooprofilattico Sperimentale del Piemonte, Liguria e Valle d’Aosta, Via Bologna 148, 10154 Torino, Italy; marzia.pezzolato@izsplv.it (M.P.); elena.biasibetti@izsplv.it (E.B.); raffaella.spedicato@izsplv.it (R.S.); serena.robetto@izsplv.it (S.R.)

**Keywords:** otter, *Lutra lutra*, gallbladder, carcinoma, neoplasm, metastatic

## Abstract

An adult female Eurasian otter (*Lutra lutra*), introduced with another subject in the National Park of Gran Paradiso (Aosta Valley Region, Italy), was found dead. The necropsy found a mass involving mainly the gallbladder walls with other multicentric masses in the liver and pancreas. In addition to these, through the histological examination, other nodules were detected in the pancreas, with structure similar to gallbladder neoplasm. Histopathology diagnosed it as neoplasia composed of epithelioid cells, forming lobules of tubules and pseudoacini, with a very low mitotic count, discrete cellular pleomorphism, and prominent fibrous stroma. Neoplastic cells demonstrated positive immunoreactivity for cytokeratin and negative immunoreactivity for S100. Gross and histologic lesions and immunohistochemical findings were consistent with a primary gallbladder carcinoma (GBC) of metastatic type. GBC is a rare neoplasm in both humans and animals, sometimes associated with cholelithiasis and cholecystitis, with few reports in the veterinary literature especially in cattle, pigs, dogs and cats. To the best of our knowledge, this is the first report of GBC in a Eurasian otter.

## Simple Summary

A gallbladder neoplasm, with secondary nodules in the liver, spleen and pancreas, was diagnosed in a Eurasian otter (*Lutra lutra*) found dead. Gross lesions, histological and immunohistochemical findings were consistent with a primary gallbladder carcinoma (GBC) of metastatic type; to the best of our knowledge, this is the first report of GBC in a Eurasian otter.

## 1. Introduction

Otters are amphibious mammals distributed almost all over the world, except for Australia and the most remote islands; they have been classified in family Mustelidae, subfamily Lutrinae, divided into seven genera—*Aonyx*, *Enhydra*, *Hydrictis*, *Lontra*, *Lutra*, *Lutrogale*, *Pteronura*—and 13 species. The European otter (*Lutra lutra*), a medium-sized (about 120 cm long) animal, lives in a very wide geographical area ranging from the Iberian Peninsula to Japan, including some North African countries such as Morocco, Tunisia and Algeria. Woodlands along rivers and lakes are its natural habitat and its diet consists mainly of freshwater fish (stickleback, brown trout, eel, and bullhead). When freshwater fish are less available, as also exhibited by other generalist predators, otters switch to alternative prey like amphibians (predominantly common frog), waterfowl, crayfish, and they even can eat water insects and vegetables [1,2]. Since the end of the 1950s, the number of Eurasian otter has suffered a sharp decline mainly caused by numerous anthropogenic factors (pollution, poaching, habitat destruction). As a result, the species, already protected in Europe by the Berne Convention and Directive Habitat (92/43/CEE), has most recently been assessed for the IUCN Red List of Threatened Species in 2023 and listed as Near Threatened (NT) worldwide [3,4,5]. In Italy, as in some countries of Asia (India, Pakistan, Bangladesh, Myanmar, and Thailand), it is classified as “Endangered” (EN), with an estimated population of about 800–1000 individuals mostly located in some central-southern regions (Campania, Molise, Calabria, Basilicata and Puglia) [6]. In recent years, thanks to reintroduction projects, the otter has reappeared in some northern regions including Valle d’Aosta, Trentino (likely introduction from Austria) and Friuli Venezia Giulia (probable introduction from Slovenia) [7]. Similar to domestic animals, among the otters or Lutrinae, especially in the sea otter (*Enhydra lutris*) and river otter (*Lontra canadensis*), different types of neoplasms are described. The assessment of the bibliographic oncological reports of the last 20 years shows a clear prevalence of hematopoietic system involvement, with lymphomas (n = 13) of various types and sites [8,9,10,11,12,13,14,15,16,17,18], followed by leiomyomas (n = 5) [19,20,21], malignant melanomas (n = 3) [21,22,23], squamocellular carcinomas (n = 3) [21,24], fibrosarcomas (n = 3) [17,18], pheochromocytomas (n = 2) [19,25], pancreatic adenomas (n = 2) [21,26], pancreatic adenocarcinomas (n = 2) [21,27], hepatocellular adenomas (n = 2) [21,28], splenic sarcomas (n = 2) [21] and hemangiosarcomas (n = 2) [21]. Single cases are described for mammary gland adenoma and adenocarcinoma [21,22], mammary malignant spindle cell tumor [21], hepatocellular carcinoma [21], biliary adenoma and carcinoma [19,21], thymoma [29], thyroid adenoma and carcinoma [21,30], parathyroid adenoma [21], omental neuroendocrine carcinoma [21], renal carcinoma [21], chondrosarcoma [17], osteosarcoma [31], basal cell carcinoma [32], ovarian teratoma [33], malignant seminoma [34], leydigoma [21], Sertoli cell tumor [21], bronchoalveolar adenoma and carcinoma [21], soft tisue sarcoma [21], and choroid plexus carcinoma [21]. In order to contribute to the knowledge of the neoplastic processes of the Eurasian otter, for which there are few oncological reports—unlike for other otter species—we describe a carcinoma of the gallbladder (GBC) in an adult female of *Lutra lutra*, living in the Gran Paradiso National Park in North-Western Italian Alps. The case, being the first report of such a tumor in this species, adds a new entry to the causes of mortality among European otters. This contributes to the conservation of endangered animals and also to the field of comparative oncology.

## 2. Case Description

**Figure 1:**
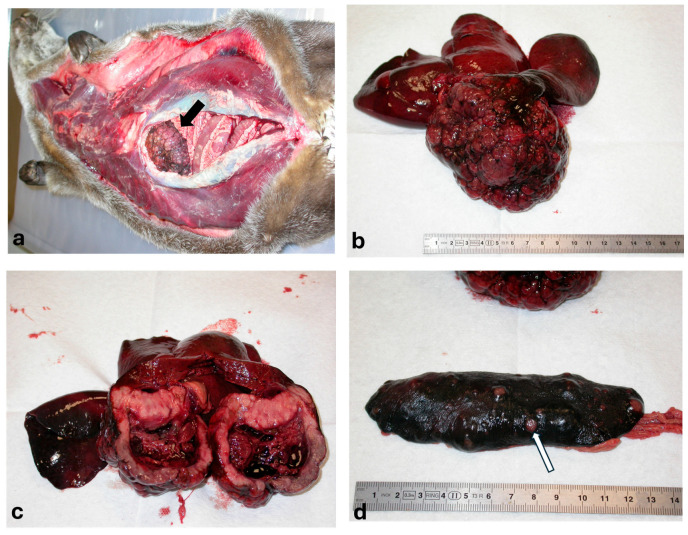
Gallbladder carcinoma—Otter (*Lutra lutra*). (**a**) Parahepatic mass at the opening of abdominal cavity (arrow); (**b**) Cauliflower neoplasia corresponding to gallbladder; (**c**) Section of neoplasm involving the gallbladder walls throughout; (**d**) Metastatic nodules of gallbladder neoplasm in the spleen (arrow).

A recently deceased female adult otter, introduced in a protected area of Gran Paradiso National Park (Valle d’Aosta Region, Italy) about two years prior to death, was found and submitted for necropsy. The animal was in good physical condition; upon opening the carcass (Figure 1a), the gallbladder appeared as a dark-red cauliflower-like mass of about 8–10 cm in diameter (Figure 1b); on sectioning, no stones were detected in the lumen and the wall was markedly thickened and extensively infiltrated by lobules of firm, waxy and hard tissue with focal intraluminal projections (Figure 1c). Much less prominent nodular masses, having a similar appearance to gallbladder neoplasm and measured approximately 0.5 cm in diameter, were observed in the splenic (Figure 1d) and hepatic parenchyma. In addition to the macroscopically visible neoplastic lesions, all internal organs were submitted for histological examination. Tissues were fixed in 10% neutral buffered formalin, trimmed, routinely processed, embedded in paraffin blocks and cut at 3 +/− 5 µm slices. All sections were stained with haematoxylin and eosin and microscopically analyzed at different magnifications by the Olympus BX60 microscope and image captured by DP-Soft Version 2.1 software.

To confirm the diagnosis, additional sections were cut on polarized slides and underwent immunohistochemical characterization. Primary antibodies directed against pan-cytokeratin (heat-induced epitope retrieval/HIER high pH, 1:50 dilution, AE1⁄AE3, mouse monoclonal; Dako) and S100 (HIER pH 6, 1:400 dilution Polyclonal Dako) were used. After deparaffinizing and rehydrating, HIER was performed with TriS-EDTA buffer, pH9-pH6 (S110), for 20 min at 97 °C in a water bath. Afterwards, the slides were treated with 3% hydrogen peroxide for 30 min, followed by two PBS washings and BSA incubation for 30 min. Each primary antibody was incubated for 30 min at room temperature. Then sections were covered with secondary antibody MACH 2 Universal HRP-Polymer Rabbit/mouse and incubated for 20 min at room temperature. The EnVision Peroxidase Dual Link System detection system (Dako, K4063) and DAB (3,3 diaminobenzidine tetrahydrochloride; Sigma, St. Louis, MO, USA) as chromogen were applied for 10 min. Then, sections were counterstained with Mayer’s hematoxylin for 2 min and assembled according to routine methods. Each experimental run incorporated control specimens, including a positive control (a canine melanoma to confirm S100 antibody reactivity and a canine carcinoma to confirm cytokeratin antibody reactivity) and a negative control, obtained by omitting the primary antibody during the labelling process, to assess non-specific background staining.

**Figure 2:**
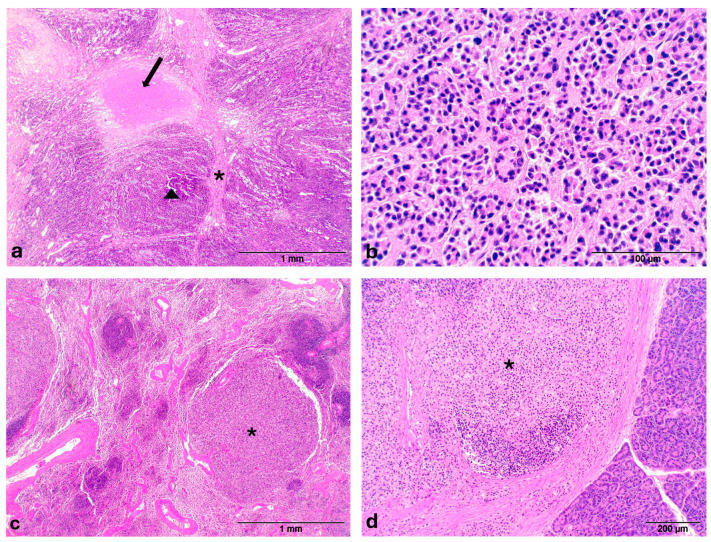
Gallbladder carcinoma—Otter (*Lutra lutra*). (**a**) Prominent fibrous trabeculae (asterisk) divide the neoplasia into lobules (triangle), occasionally associated with necrotic area (arrow); (**b**) Acini and pseudo-tubules supported by a fine fibrovascular stroma, characterized by absence of mitotic activity, not-evident mucinous secretion and moderate anisocytosis; (**c**) Metastatic nodule in the spleen (asterisk); (**d**) Metastatic nodule in the pancreas (asterisk).

**Figure 3:**
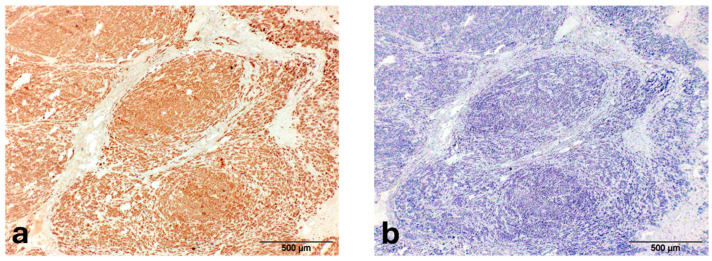
Gallbladder carcinoma—Otter (*Lutra lutra*) (**a**) GBC: Diffuse cytoplasmic positivity for cytokeratin; (**b**) GBC: S100 negativity.

The microscopic evaluation revealed in the gallbladder a neoplasia characterized by lobules, separated by thick fibrous connective trabeculae (Figure 2a) and consisting of acinar and pseudotubular structures, generally not associated with an evident mucinous secretion, supported by a fine fibrovascular stroma; neoplastic cells were characterized by discrete anisocytosis, cytoplasm intensely eosinophilic, nuclei with chromatin finely dispersed and peripherally thickened, single voluminous nucleolus and mitotic rate of <2/10HPF (Figure 2b); in the context of neoplastic lobules large necrotic areas were present (Figure 2a). The nodules in the liver (Figure S1) and spleen (Figure 2c) were characterized by irregular peripheral fibrous capsule (more prominent in the liver), structure and cells similar to primary neoplasm, occasionally arranged in more solid sheets without lumina (Figure S2), and absence of necrotic foci. Neoplastic growth with the same features of hepatic and splenic masses was also detected in the pancreas (Figure 2d). Except for the liver parenchyma, showing intracellular cholestasis and diffuse lipidosis, histological lesions different from a neoplastic change were not observed in all internal organs. Regarding the immunohistochemical pattern, neoplastic cells showed a strong cytoplasmic positivity for cytokeratin while they were negative for S100 (Figure 3a,b). Given the histological findings (glandular structure and low mitotic index) and immunohistochemical pattern (cytokeratin-positivity), in addition to the presence of similar neoplastic nodules of smaller size in the liver, spleen and pancreas, the neoplasm—according to Cullen J.M [35]—has been classified as a malignant neoplasm of epithelial origin comparable to a primary gallbladder carcinoma (GBC) of metastatic type.

In the absence of metastases to other vital organs like lungs and the limited secondary nodules in pancreas, spleen and liver, the death of the animal is probably attributable to the effects of the neoplastic process inside the gallbladder onto the hepatic parenchima (probably failure of the biliary system such as duct blockage with consequent hepatocytes damage).

## 3. Discussion

A neoplasm involving the gallbladder generally poses a problem of differential diagnosis between the three neoplasms commonly recognized in this anatomical district among the animals affected (cattle, pig, dog and cat) [35]. These are adenoma, carcinoma and neuroendocrine carcinoma (or carcinoid), but—given that GBC is essentially classified as extrahepatic cholangiocellular carcinoma—cholangiocarcinoma arising within intrahepatic biliary system should also be considered. Adenoma was excluded for the presence of metastases to the liver, spleen and pancreas; neuroendocrine carcinoma for the low mitotic activity and immunohistochemical characteristics (neuroendocrine carcinomas are identifiable immunohistochemically first for positivity to S100 as well as to chromogranin A and neuron-specific enolase). Regarding cholangiocellular carcinoma, an origin from bile ducts was not consistent with the absence of mucinous secretion and the low mitotic count, respectively abundant and high in typical intrahepatic cholangiocarcinomas [30]. The neoplasm observed in our case was consistent with a primary GBC for the following characteristics: (a) positivity for cytokeratin and negativity for S100 (this last result is reliable considering both the genomic loci encoding S100 proteins is highly conserved in mammals [36] and the same primary antibody we used tested positive for other neoplasm of otter, e.g., malignant melanoma [22]); (b) a structure similar to the GBCs described in the domestic animals, with acinar and tubular structures separated by thin fibrous stroma and a low mitotic count. As observed in some species such as dogs, despite the scarce mitotic activity and the moderate degree of differentiation, the behavior of GBCs is rather aggressive with a tendency to expand locally and then metastasize into the adjacent hepatic parenchyma and later on the serosal surfaces of the abdominal cavity, lymph nodes and lung [35].

GBC is quite rare in domestic animals [35]. In humans, it is the most common malignant neoplasm of the biliary tract worldwide and its risk factors have been statistically identified and listed as follows: demographic factors (advanced age, female gender, obesity, South America/India/Pakistan/Japan and Korea origin, genetic predisposition), gall bladder pathology/abnormalities (cholelithiasis, porcelain gall bladder, polyps, congenital biliary cysts, pancreaticobiliary anomalies), exposures (heavy metals, medication such as methyldopa, isoniazid and estrogen, smoking) and infections (*Salmonella*, *Helicobacter*). Cholelithiasis is considered the main risk factor, with an 8.3 higher risk than the general population [37,38]. It is postulated that GBC may be intimately associated with large or numerous cholesterol gallstones that in the first instance may interfere with the mechanical functioning of the gallbladder. The size as well as the number of stones may contribute significantly to the promotion of filling defects of the gallbladder that may cause chronic mechanical damage to the gall bladder mucosa [39,40].

Similar to humans, even if the studies are more limited in animals (and completely missing in otters), it has been observed also in aged pigs that chronic cholecystitis associated with gallstones and/or bacterial infections may contribute to metaplastic changes and development of gallbladder neoplasms [41] while cholecystoliths are not commonly associated with GBC in dogs [42].

Excluding the female gender and the advanced age, for the GBC detected in this otter we have not identified other risk factors recognized in humans. Cholelythiasis as cholecystitis, which as mentioned above, mainly predisposes humans to GBC onset, is not frequently reported in otters and in any case never associated with neoplasms in the gallbladder [43,44]. The absence of stones and inflammatory processes supports the hypothesis that in this animal GBC was a spontaneous neoplasm not secondary to other pre-existing pathological conditions. Further research needs to demonstrate if the neoplasm was caused by exposure to carcinogenic chemical agents. However, it is possible to rule them out because there were not any industrial activities or other sources of pollution in the mountain ecosystem where the otter lived.

As with other wild animals, studies conducted in England, Germany and Italy indicate that the leading causes of mortality in European otters are traumatic events (e.g., road traffic accidents, bite wounds, etc.), accidents being mostly frequent in winter, and dog bites in summer. Infectious and non-infectious diseases (e.g., parasitic, viral or bacterial processes) are observed less frequently and typically as incidental findings in animals that died due to trauma [9,45,46]. Neoplasms are even more rarely reported; as noted in the Introduction, neoplasms in the European otter (*Lutra lutra*)—unlike the more frequently studied sea otter and river otter—are scarce and currently include only two malignant melanoma (one with concurrent trichoblastoma and mammary adenoma) [22,23], two lymphomas [8,9], one hepatocellular adenoma [28] and one undefined metastatic neoplasia in an Italian specimen [47].

## 4. Conclusions

Cancer is still a rare disease—generally not age related—in wildlife compared to captive animals; however, in recent years, also due to greater attention to the causes of mortality among the free-ranging species in a One Health perspective and biodiversity conservation, frequency of oncological reports is increasing. Toxin exposures, pollution, oncogenic pathogens, stress, immunosuppression and genetic anomalies can all contribute to neoplasm development in wild animals. Wild species at the interface between humans and the environment are growing, making wildlife sentinel for both animal and human health and so offering novel insights into potentially unique non-age-related mechanisms of carcinogenesis across species [48].

In this context, the GBC described in our case—the first such report in this species—adds new knowledge on neoplasm pathology in this mustelid and, more broadly, contributes to comparative oncology in wildlife.

## Data Availability

Individual raw data collected are available on request from the corresponding author.

## Supplementary Materials

The following supporting information can be downloaded at: https://www.mdpi.com/article/10.3390/ani15172484/s1. Figure S1: Gallbladder carcinoma—Otter (*Lutra lutra*). Metastatic nodule of GBC in the liver (asterisk). Figure S2: Gallbladder carcinoma—Otter (*Lutra lutra*). Metastatic nodule of GBC in the liver arranged in solid sheets without lumina.
