# Supplementary material for: Gallbladder Carcinoma in a Eurasian Otter (Lutra lutra)
Source: Animals (Basel). 2025 Aug 24;15(17):2484. doi: 10.3390/ani15172484 (PMC12427233; doi:10.3390/ani15172484)
Supplement: Supplementary file 1 [file animals-15-02484-s001.zip › animals-3760411-supplementary.pdf]

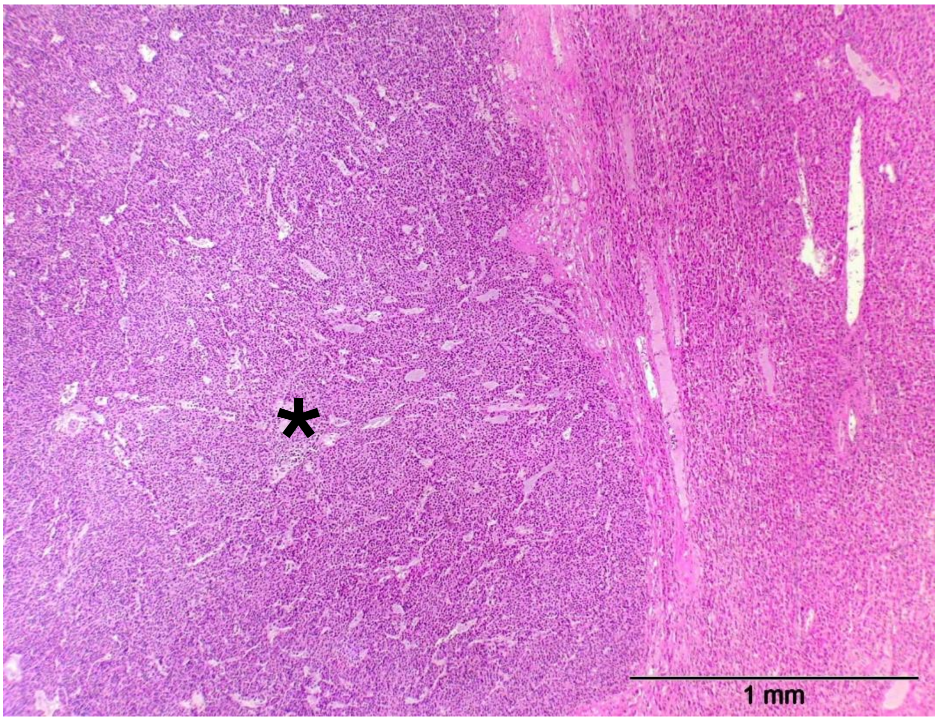

Figure S1: Gallbladder Carcinoma - Otter (*Lutra lutra*). Metastatic nodule of GBC in the liver (asterisk).

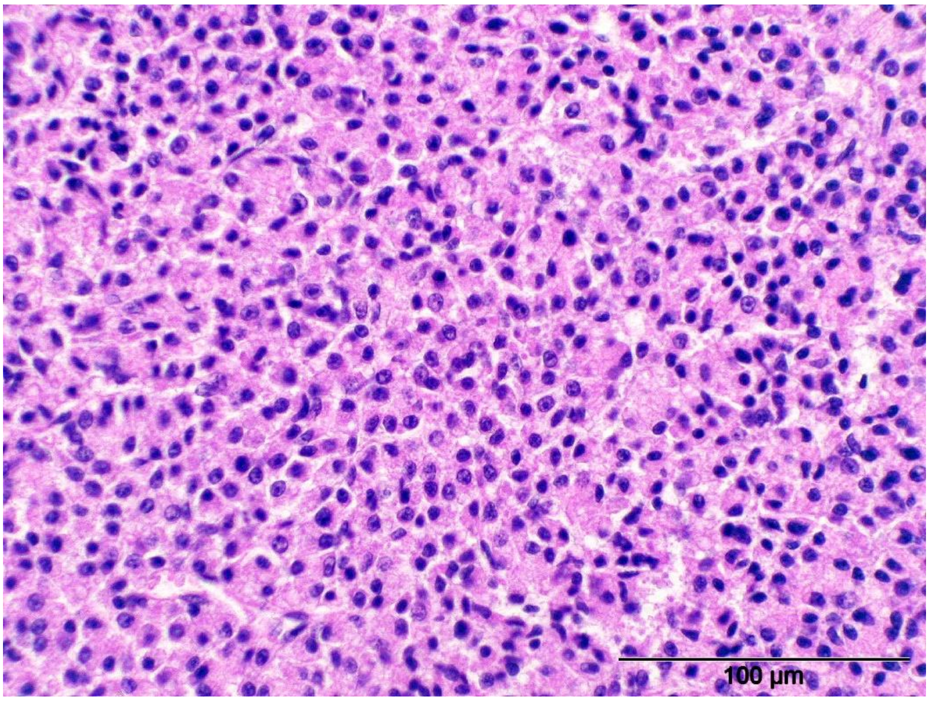

Figure S2: Gallbladder Carcinoma - Otter (*Lutra lutra*). Metastatic nodule of GBC in the liver arranged in solid sheets without lumina.
